# DMGAT: predicting ncRNA-drug resistance associations based on diffusion map and heterogeneous graph attention network

**DOI:** 10.1093/bib/bbaf179

**Published:** 2025-04-19

**Authors:** Tingyu Liu, Qiuhao Chen, Renjie Liu, Yuzhi Sun, Yadong Wang, Yan Zhu, Tianyi Zhao

**Affiliations:** School of Medicine and Heath, Harbin Institute of Technology, 150000, Nangang District, Xidazhi Street No. 90, Harbin, China; Zhengzhou Research Institute, Harbin Instituteof Technology, 150000, Nangang District, Xidazhi Street No. 90, Harbin, Heilongjiang, China; Zhengzhou Research Institute, Harbin Instituteof Technology, 150000, Nangang District, Xidazhi Street No. 90, Harbin, Heilongjiang, China; School of Computer Science and Technology, Harbin Institute of Technology, 150000, Nangang District, Xidazhi Street No. 90, Harbin, Heilongjiang, China; School of Computer Science and Technology, Harbin Institute of Technology, 150000, Nangang District, Xidazhi Street No. 90, Harbin, Heilongjiang, China; College of Veterinary Medicine, Northeast Agricultural University, 150038, Xiangfang District, Changjiang Road No. 600, Harbin, China; School of Medicine and Heath, Harbin Institute of Technology, 150000, Nangang District, Xidazhi Street No. 90, Harbin, China; Zhengzhou Research Institute, Harbin Instituteof Technology, 150000, Nangang District, Xidazhi Street No. 90, Harbin, Heilongjiang, China

**Keywords:** ncRNA-drug resistance association prediction, diffusion map, graph convolution network, graph attention network

## Abstract

Non-coding RNAs (ncRNAs) play crucial roles in drug resistance and sensitivity, making them important biomarkers and therapeutic targets. However, predicting ncRNA-drug associations is challenging due to issues such as dataset imbalance and sparsity, limiting the identification of robust biomarkers. Existing models often fall short in capturing local and global sequence information, limiting the reliability of predictions. This study introduces DMGAT (diffusion map and heterogeneous graph attention network), a novel deep learning model designed to predict ncRNA-drug associations. DMGAT integrates diffusion maps for sequence embedding, graph convolutional networks for feature extraction, and GAT for heterogeneous information fusion. To address dataset imbalance, the model incorporates sensitivity associations and employs a random forest classifier to select reliable negative samples. DMGAT embeds ncRNA sequences and drug SMILES using the word2vec technique, capturing local and global sequence information. The model constructs a heterogeneous network by combining sequence similarity and Gaussian Interaction Profile kernel similarity, providing a comprehensive representation of ncRNA-drug interactions. Evaluated through five-fold cross-validation on a curated dataset from NoncoRNA and ncDR, DMGAT outperforms seven state-of-the-art methods, achieving the highest area under the receiver operating characteristic curve (0.8964), area under the precision-recall curve (0.8984), recall (0.9576), and F1-score (0.8285). The raw data are released to Zenodo with identifier 13929676. The source code of DMGAT is available at https://github.com/liutingyu0616/DMGAT/tree/main.

## Introduction

Non-coding RNAs (ncRNAs) play important roles in cells’ development, differentiation, and apoptosis processing. Numerous studies have shown that ncRNAs are extensively involved in human pathological pathways. As biomarkers, they offer new targets for treating diseases such as cancer [1–3]. Non-coding RNAs, such as microRNAs (miRNAs), circular RNAs (circRNAs), long non-coding RNAs (lncRNAs), and Piwi-interacting RNAs (piRNAs), have garnered significant interest from researchers. Recent studies suggest that ncRNAs are involved in various aspects of tumor cell drug resistance, including epithelial–mesenchymal transition, DNA repair, drug efflux and metabolism, and cell cycle progression, highlighting the potential significance of ncRNAs in drug therapy [4, 5]. Different ncRNAs have different functions according to their length and structure. miRNAs are short regulatory biomolecules involved in post-transcriptional regulation of gene expression. Compared to linear miRNAs, circRNAs [6] are more stable and may function as carriers or scaffolds [7]. They regulate protein function by acting as microRNA or protein inhibitors, or they can be translated to perform important biological functions [8, 9]. lncRNAs can play a role in regulating synergistic proteins. In contrast, piRNAs have been studied relatively less. Extending across 24–35 nucleotides [10–12], piRNAs interact with proteins of the Piwi subfamily and are essential for the suppression of transposable elements, protection of the genome, and histone modification, among other roles, influencing gene expression and primarily suppressing transposon activity. There are synergistic interactions between different RNAs. For example, lncRNAs can act as molecular sponges for miRNAs, regulating the expression of their target genes.

The increasing volume of studies connecting abnormal ncRNA expression to various drugs highlights the potential of these ncRNAs as valuable diagnostic markers and therapeutic targets. More specifically, the antagonistic interactions between certain ncRNAs and specific types of drugs can affect protein expression, which in turn influences human metabolism. For example, miR-181a interacts with drugs by modulating molecular pathways involved in glioblastoma drug resistance, potentially altering the tumor’s responsiveness to treatments [13]. One study shows miR-181a influences drug efficacy by regulating key molecular mechanisms related to glioblastoma drug resistance, potentially affecting treatment outcomes [14]. miR-181a modulates drug resistance in glioblastoma by influencing molecular pathways that control tumor sensitivity to chemotherapy, potentially impacting treatment success [15].

With the decreasing cost and advancements in sequencing technology, many databases now offer a wealth of resistance and sensitivity associations between ncRNAs and drugs, including experimentally validated and predicted associations. ncDR [16] database has collected 5864 experimental verified resistance relationships between 1039 ncRNAs (162 lncRNAs and 877 miRNAs) and 145 compounds from around 900 published studies. It used in a lot of ncRNAs association prediction studies such as LRGCPND [17], RDRGSE [18] and GSLRDA [19]. NoncoRNA [20] provide an all-encompassing database for searching drug resistance/sensitivity-related ncRNAs in different human cancers. ncRNADrug [21] is a comprehensive and integrated database that collected manually curated and predicted resistance/sensitivity associations between ncRNAs and drugs. These databases provide a wealth of knowledge for understanding the associations between ncRNAs and drugs in humans.

To date, numerous ncRNA-drug associations have been validated through experimental biological methods. However, progress in this field has been constrained by the significant time and resource investments required for such validation [22, 23]. In response to these limitations, researchers have developed various computational algorithms to efficiently explore and map ncRNA-drug networks. For example, the NTSHMDA [24] model constructs a heterogeneous network integrating disease and microbe similarities with known associations, using a weighted random walk algorithm based on network topology. In KATZHMDA [25], a heterogeneous network combines multisource miRNA and disease similarity networks with known associations, predicting links via the KATZ algorithm. SDLDA [26] applies singular value decomposition and deep learning to predict lncRNA-disease associations. AE-RF [27] combines deep autoencoder and random forest for circRNA-disease predictions. ABHMDA [28] uses k-means clustering to select reliable negative samples and an adaptive boosting classifier for microbe-disease predictions. DMFCDA [29] employs deep matrix decomposition with a fully connected projection layer to extract latent circRNA-disease features, feeding them into a neural network. In DMFMDA [30], one-hot encoded microbe and disease data is transformed to low-dimensional vectors through an embedding layer, with predictions made via matrix decomposition in a neural network.

Although the mentioned methods have demonstrated good performance, there is still a room to enhance the processing of mining the feature of ncRNAs and drugs using more comprehensive information. On one hand, current methods like NTSHMDA and DMFMDA, do not take into account the sequence information of RNA and drugs, relying solely on association matrices to derive features. This approach loses a lot of valuable information contained within the sequences. On the other hand, existing models focus only on resistance while treating all other associations as unknown. This means that all unknown associations are excluded from the parameter iteration process, overlooking the presence of sensitivity associations among them.

To overcome these limitations, we introduced the diffusion map and heteregeneous graph attention network (DMGAT) model, which incorporates an attention-based graph convolutional network along with sensitivity associations in diffused space using diffusion map. Diffusion map can make ncRNA or drug features more continuous in the manifold space, while GAT graph neural network utilizes attention mechanisms to fully capture the association features between ncRNA and drugs. The key contributions of our work are outlined as follows:

- We introduce DMGAT, a novel deep learning model that integrates diffusion maps with graph convolutional and attention networks for accurate prediction of ncRNA-drug associations.
- DMGAT employs the word2vec technique to embed ncRNA sequences and drug SMILES while constructing a heterogeneous network that combines sequence and Gaussian interaction profile similarities.
- Our approach tackles data imbalance and sparsity by incorporating sensitivity associations and using a random forest classifier to select reliable negative samples.

Our source code and data are available on github (https://github.com/liutingyu0616/DMGAT/tree/main).

## Materials

### Dataset

The manually curated ncRNA-drug associations datasets NoncoRNA [20], ncDR [16] and ncRNADrug [21] are collected as benchmark dataset used in our work. We used the resistance associations in NoncoRNA and ncDR dataset, the sensitivity associations are from ncRNADrug dataset.

- NoncoRNA
  NoncoRNA: containing 8233 ncRNA-drug resistance associations between 5568 ncRNAs and 154 drugs in 134 cancers [20]. The 2020 February version is used in our work, which is released at http://www.ncdtcdb.cn:8080/NoncoRNA.
- ncDR
  ncDR is an aggregated dataset that contains manually curated verified and predicted ncRNA-drug associations. We used 2016 June version of ncDR dataset, which involves 145 drugs and 1039 ncRNAs (877 miRNAs and 162 lncRNAs) from around 900 published literatures [16]. The dataset is public releases at https://www.mdpi.com/1422-0067/22/19/10508.
- ncRNADrug
  It comprises ncRNAs linked to drug resistance, along with ncRNAs that are drug targets, experimentally confirmed and computationally predicted. Regarding experimentally validated entries, ncRNADrug includes 29 551 resistance records between 9195 ncRNAs (2248 miRNAs, 4145 lncRNAs, and 2802 circRNAs) and 266 drugs [21]. Additionally, it contains 32 969 records involving 10 480 ncRNAs (4338 miRNAs, 6087 lncRNAs, and 55 circRNAs) that are targeted by 965 drugs.

We only selected experiment verified associations from those three datasets. We refined the dataset by eliminating redundant and ambiguous associations and ones where a lncRNA or miRNA is linked to only a single drug resistance binding from NoncoRNA and ncDR. Additionally, we choose the sensitivity associations and removing the redundant associations from ncRNADrug dataset. After data preprocessing, we got 2693 resistance associations and 408 sensitivity associations between 622 ncRNAs (41 lncRNAs and 581 miRNAs) and 121 drugs. The dataset can be denoted as,


(1) \begin{align*}& \mathbb{S}=\mathbb{S}^{\text{res}}+\mathbb{S}^{\text{sen}}+\mathbb{S}^{\text{unknown}}\end{align*}

where $\mathbb{S}^{\text{res}}$ represents the resistance associations, which contains 2689 ncRNA-drug resistance entries, $\mathbb{S}^{\text{sen}}$ represents the sensitivity associations, which contains 408 ncRNA-drug sensitivity associations. $\mathbb{S}^{\text{unknown}}$ represents the associations which not be verified experimentally, the number of $\mathbb{S}^{\text{unknown}}$ associations is 72576. Known associations account for 3.7% of the total associations.

## Methodology

**Figure 1:**
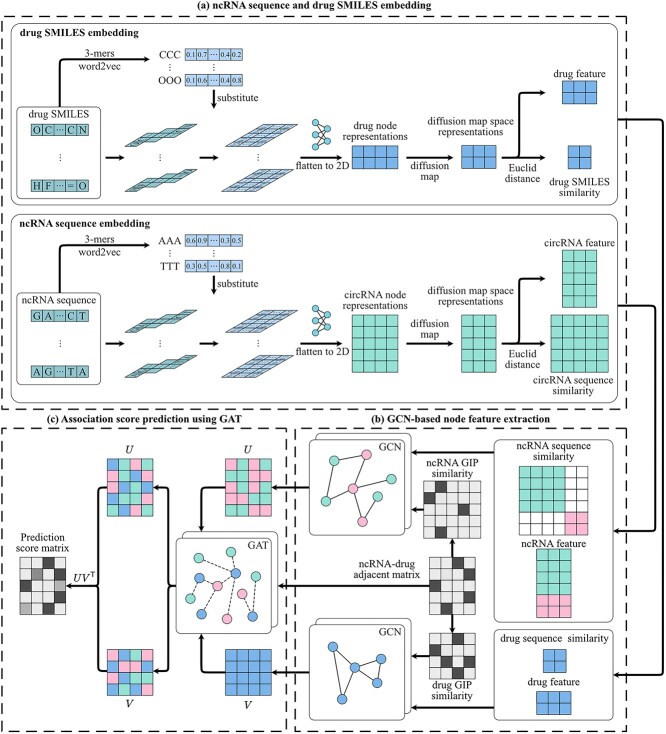
The flowchart of DMGAT. (a) ncRNA and drug node embedding and similarity calculation. Split sequence of ncRNA and SMILES of drug into several 3-mers, and then replaced by their corresponding feature vectors derived from word2vec. Flatten the feature to 2D using a deep learning network. Using diffusion map to reduce the dimension of feature. Sequence similarity obtained by calculating the Euclid distance of their feature. (b) Feature extraction using GCN. Contracting two separate homogeneous graph convolution networks to extracting ncRNA and drug feature respectively based on their diffusion map feature and similarity. (c) Predicting resistance association score using graph attention network. Combining feature of ncRNA and drug based on attention mechanism, multiply two feature matrices to get the predicted adjacency matrix.

We proposed a ncRNA-drug association predictor DMGAT based on graph attention network and graph attention network in diffusion map space. Random forest classifier and sensitivity associations were introduced to select reliable negative association while training to tackle imbalance dataset problem. the workflow of DMGAT is shown in Fig. 1. There are three mainly steps, (i) embedding ncRNA sequence and drug SMILES using word2vec technique, using diffusion map to reduce the dimension, computing the similarity in diffusion map space. (ii) Extracting the ncRNA and drug feature using graph convolution network, respectively. (iii) Predicting association using graph attention network.

### ncRNA sequence and drug SMILES embedding

In most previous studies, the combination of the row and column of adjacent matrix between ncRNAs and drugs was commonly regarded as the feature of each association. However, the adjacent matrix is sparse, which could not describe the difference of each ncRNA-drug resistance pair accurately. Meanwhile, it will lose the information implied in the sequence of ncRNAs and SMILES (simplified molecular input line entry system) of drugs [31]. SMILES is a notation used to represent the structure of drugs. It encodes the molecular structure of a compound by using specific characters to denote atoms, bonds, and connectivity.

#### Word2Vec encoding

To avoid the sparse problems of feature matrix, we introduced a widely used long sequence embedding technique in natural language model processing field, which is word2vec [32]. The development of word2vec technique is applied in the genism library [33], which utilizes continuous bags of words (CBOW) by default. We split each sequence into 3-mers, ncRNA sequences and drug SMILES, and used the feature of these 3-mers to encapsulate the whole sequence’s feature. This method not only can represent the information implied in local sequence, but also could consider the positional relationship between each local 3-mers, which could reflect the patterns inherent to the sequence and keep the order information.

The sequences information of lncRNAs are obtained from LncBook 2.0 (https://ngdc.cncb.ac.cn/lncbook/home) [34]. The sequences of miRNAs are obtained from miRbase (http://mirbase.org/) [35]. The SMILES of drugs are obtained from DrugBank (https://go.drugbank.com/), which is a “gold standard” knowledge base for drug-related information, including drug-target interactions and other pharmaceutical data [36].

After word2vec encoding, each feature of ncRNA and drug is a 2D array, so the feature of each type of ncRNA is a combination of many 2D arrays, that is a 3D array. For subsequent data processing, it is necessary to further encode the 3D array into a 2D array. Here, a fully connected neural network layer is used to reduce the features from 3D to 2D. The optimization direction of the loss function is to make the variance of the compressed 2D array as large as possible. This setting aims to make the features after linear compression more dispersed in the feature space and reflect differences between different ncRNAs and drugs.

#### Diffusion map

Given that RNA mutates only a few bases at a time, the characteristic of RNA may be continuously changing along a certain path. For a drug, it is assumed that drugs with similar SMILES have similar functional properties, so diffusion mapping method is used to compress the characteristics of ncRNAs and drugs. Diffusion maps [37] are a powerful tool for encoding long sequences by capturing the intrinsic geometric structure of high-dimensional data. This technique constructs a diffusion process based on the pairwise similarities between sequences, which allows for the preservation of both local and global relationships in the sequence of ncRNAs and drugs [38]. Unlike linear dimensionality reduction methods like PCA, diffusion maps excel at identifying non-linear patterns and having the ability to capture continuous changing features, which are critical in biological datasets where the relationships between features are often complex. The use of diffusion maps for sequence encoding ensures robustness to noise, preserves the biological manifold structure, and provides an effective means for dimensionality reduction, facilitating further downstream analysis [39]. The number of eigenvectors to compute in the diffusion map for lncRNA, miRNA, and drug are 9, 150, and 40, respectively, which are a quarter of their feature dimension approximately. Scale parameter epsilon detected by an algorithm by Berry, Harlim, and Giannakis. Normalization parameter alpha is set to 1, which ensures probability conservation, using ball tree algorithm for nearest neighbor search.

A full connected layer was used to extract the feature from diffusion map space in order to unify the dimension of each kind of ncRNA and drug.

### ncRNA-drug adjacency network construction

The adjacency matrix shows if there is a resistance association between each ncRNA and drug. It can be denoted as,


(2) \begin{align*}& \boldsymbol{A} =\left[\begin{array}{cccc} a_{1,1} & a_{1,2} & \cdots & a_{1, n} \\ a_{2,1} & a_{2,2} & \cdots & a_{2, n} \\ \vdots & \vdots & \ddots & \vdots \\ a_{m, 1} & a_{m, 2} & \cdots & a_{m, n} \end{array}\right]\end{align*}

where $m$ is the number of ncRNAs, $n$ is the number of drugs. $a_{ij}=1$ if there is a resistance association between the $i$th ncRNA and the $j$th drug, and $a_{ij}=0$ otherwise.

#### ncRNA similarity matrix

The similarity between ncRNAs is obtained from the sequence of each ncRNA and the adjacency matrix, which can be denoted as,


(3) \begin{align*}& \boldsymbol{PS}_{\text{seq}}\in\mathbb{R}^{m\times m}\end{align*}

The sequence similarity is obtained by computing the Euclid distance between each ncRNA in diffusion map space, which can be represented as,


(4) \begin{align*}& S_{r}^{\text{seq}}(r_{i}, r_{j})=\operatorname{Euclid}(r_{i}, r_{j})\end{align*}

where $r_{i}$, $r_{j}$ are the $i$th and $j$th ncRNA, respectively.

The Gaussian interaction profile (GIP) kernel similarity has been widely utilized to assess the similarity between two nodes in the prediction of ncRNA-drug associations [38, 40]. This implies that ncRNAs with similar profiles tend to exhibit similar interaction patterns in drugs, and the reverse is also true. It captures the similarity between these entities by creating a Gaussian distribution of interaction profiles based on available interaction data. The GIP kernel similarity between $i$th and $j$th ncRNA is as follows [37],


(5) \begin{align*}& S_{r}^{\text{GIP}}(r_{i}, r_{j})=\exp \left(-\lambda_{r}\|\boldsymbol{A}(r_{i},)-\boldsymbol{A}(r_{j},)\|^{2}\right)\end{align*}

where $\boldsymbol{A}(r_{i},)$ and $\boldsymbol{A}(r_{j},)$ are the $i$th and $j$th row vector of the adjacency matrix $\boldsymbol{A}$, $\lambda _{r}$ is the kernel width coefficient, which is defined as,


(6) \begin{align*}& \lambda_{r}=\frac{1}{\frac{1}{N_{r}} \sum_{k=1}^{N_{r}}\left\|\boldsymbol{A}(r_{k}, )\right\|^{2}}\end{align*}

where $N_{r}$ is the total number of lncRNAs and miRNAs, $\boldsymbol{A}(r_{k},)$ is the $k$th row vector of the adjacency matrix $\boldsymbol{A}$.

The combined integrated similarity between ncRNAs is calculated by taking the average of the sequence similarity and GIP kernel similarity,


(7) \begin{align*}& \boldsymbol{S}_{r}=\frac{\boldsymbol{S}_{r}^{\text{seq}}+\boldsymbol{S}_{r}^{\text{GIP}}}{2}\end{align*}

#### Drug similarity matrix

The similarity between drugs is obtained from the SMILES of each drug and the adjacency matrix. The way of calculating the SMILES similarity between drugs is similar to that of ncRNAs, which can be denoted as,


(8) \begin{align*}& S_{d}^{\text{seq}}(d_{i}, d_{j})=\operatorname{Euclid}(d_{i}, d_{j})\end{align*}

where $d_{i}$, $d_{j}$ are the $i$th and $j$th drug, respectively.

The GIP kernel similarity between $i$th and $j$th drug is as follows:


(9) \begin{align*}& S_{d}^{\text{GIP}}(d_{i}, d_{j})=\exp \left(-\lambda_{d}\|\boldsymbol{A}(, d_{i})-\boldsymbol{A}(, d_{j})\|^{2}\right)\end{align*}

where $\boldsymbol{A}(, d_{i})$ and $\boldsymbol{A}(, d_{j})$ are the $i$th and $j$th column vector of the adjacency matrix $\boldsymbol{A}$, $\lambda _{d}$ is the kernel width coefficient, which is defined as,


(10) \begin{align*}& \lambda_{d}=\frac{1}{\frac{1}{N_{d}} \sum_{k=1}^{N_{d}}\left\|\boldsymbol{A}(, d_{k})\right\|^{2}}\end{align*}

where $N_{d}$ is the total number of drugs, $\boldsymbol{A}(, d_{k})$ is the $k$th column vector of the adjacency matrix $\boldsymbol{A}$.

The combined integrated similarity between ncRNAs is calculated by taking the average of the sequence similarity and GIP kernel similarity,


(11) \begin{align*}& \boldsymbol{S}_{d}=\frac{\boldsymbol{S}_{d}^{\text{seq}}+\boldsymbol{S}_{d}^{\text{GIP}}}{2}\end{align*}

### Feature extraction using GCN

GCN has been widely used in different nodes feature aggregation due to its ability to capture hidden graph relations and propagate information through the network [19, 41–45], which is a type of neural network capable of efficiently extracting features from networks by aggregating information from neighboring nodes. ncRNA and drug can be regarded as node in their own graph network, their similarity matrix is the feature transition matrix while propagating feature processing [46]. The GCN takes the ncRNA, drug feature and similarity matrix as input and performs graph convolution operations to fuse their hidden features.

In GCN, the output of the lth layer is treated as the input for the $(l + 1)$th layer to capture higher dimensional features. The node embedding at the $(l + 1)$th layer is given by:


(12) \begin{align*}& \boldsymbol{H}^{l+1}=\sigma\left({\boldsymbol{D}}^{-\frac{1}{2}} {\boldsymbol{S}_{h}} {\boldsymbol{D}}^{-\frac{1}{2}} \boldsymbol{H}^{l} \boldsymbol{W}^{l}\right)\end{align*}

where $\boldsymbol{S}_{h}$ is the integrated similarity matrix obtained by Equation 7 and 11 for ncRNA and drug respectively, $\boldsymbol{D}$ is the degree matrix of $\boldsymbol{S}_{h}$, $\boldsymbol{H}^{l}$ is the ncRNA embedding of the $l$th layer, which is the concatenated feature by two kinds of ncRNAs. For the drug graph convolution network, $\boldsymbol{H}^{l}$ is the drug feature obtained from diffusion map space. $\boldsymbol{W}^{l}$ is the trainable parameter matrix. $\sigma $ is the activation function ReLU. So that the feature of ncRNAs and drugs propagated by GCN are obtained, respectively. The number of GCN layer for ncRNA and drug is 2.

### Feature extraction using GAT

The above homogeneous GCN only considers the similarity relationship between ncRNA and drug themselves, and the relationship between ncRNA and drug is integrated using GAT [47, 48]. GAT is a neural network designed to operate on graph-structured data by applying attention mechanisms to learn the importance of neighboring nodes [49]. In DMGAT, the GAT layer was used to aggregate the feature of ncRNAs and drugs based on the adjacency matrix to capture the high dimensional features of ncRNAs and drugs.

For the network GAT, the input is the ncRNA and drug feature obtained from GCN and the adjacency matrix between them. The output $(l+1)$th layer was as follows,


(13) \begin{align*}& \boldsymbol{H}_{i}^{l+1}=\sigma\left(\sum_{j \in N_{i}} \alpha_{ij} \boldsymbol{W} \boldsymbol{H}_{j}^{l}\right)\end{align*}

where $\sigma $ is the non-linear activation function ReLU. $\alpha _{i j}$ represents the normalized attention coefficient between node $i$ and its neighbor node $j$. It essentially measures the importance or relevance of node $j$ to node $i$ when aggregating information from its neighbors, which is defined as,


(14) \begin{align*}& \alpha_{i j}=\frac{\exp \left(\operatorname{LR}\left(a^{T}\left[\boldsymbol{W} \boldsymbol{H}_{i} \| \boldsymbol{W} \boldsymbol{H}_{j}\right]\right)\right)}{\sum_{k \in N_{i}} \exp \left(\operatorname{LR}\left(a^{T}\left[\boldsymbol{W} \boldsymbol{H}_{i} \| \boldsymbol{W} \boldsymbol{H}_{k}\right]\right)\right)}\end{align*}

where $N_{i}$ is the set of neighbors of node $i$, $a$ is a learnable weight vector for computing attention, LR is the activation function LeakyReLU, $\boldsymbol{W}$ is a weight matrix applied to the node features. RNA and drug obtain new integrated characteristics after GAT fusion. The number of GAT layer for ncRNA and drug is 4.

### Selecting reliable negative associations

Due to reasons such as technology or cost of sequencing, there are a lot of associations between ncRNAs and drugs unfound yet. In many studies about ncRNA-drug associations prediction tasks using graph network, unlabeled associations are treated as negative samples while training [17–19]. Considering all unlabeled examples as negative instances may lead to bias in the learning process, as these samples do not accurately reflect the true negative class. This approach can distort the dataset’s distribution and result in poor performance of the trained model. As well as introduced the imbalanced problem. So, we introduced the sensitivity associations between ncRNAs and drugs as the negative samples. Because the negative samples must be associations that are not resistant, selecting sensitivity associations that have been experimentally verified as negative samples is the most reliable method for determining negative samples. Biological experiments ensure that these associations are definitely not resistant. However, the number of sensitivity associations is significantly less than the number of positive associations, approximately one-fifth of the latter. Therefore, we use a random forest classifier with resistance associations as positive samples and sensitivity associations as negative samples. We train a random forest classifier and select the sample with the lowest score as reliable negative sample to ensure that the known sensitivity associations and predicted sensitivity associations are equal in quantity to positive samples, thus solving the issue of imbalance.

## Results

### Performance evaluation

To evaluate the performance of DMGAT systematically and objectively, the five-fold cross validation was utilized while training. The experimental confirmed ncRNA-drug resistance association set is denoted as $\mathbb{S}^{+}$. The unlabelled association set is denoted as $\mathbb{S}^{\text{U}}$. The union of verified ncRNA-drug sensitivity association set and predicted reliable sensitivity association set is denoted as $\mathbb{S}^{-}$. They can be divided into five subsets with the same size as follows,


(15) \begin{align*}& \begin{aligned} \mathbb{S}^{+} &= \mathbb{S}^{+}_{1} \cup \mathbb{S}^{+}_{2} \cup \mathbb{S}^{+}_{3} \cup \mathbb{S}^{+}_{4} \cup \mathbb{S}^{+}_{5}\\ \mathbb{S}^{\text{U}} &= \mathbb{S}^{\text{U}}_{1} \cup \mathbb{S}^{\text{U}}_{2} \cup \mathbb{S}^{\text{U}}_{3} \cup \mathbb{S}^{\text{U}}_{4} \cup \mathbb{S}^{\text{U}}_{5}\\ \mathbb{S}^{-} &= \mathbb{S}^{-}_{1} \cup \mathbb{S}^{-}_{2} \cup \mathbb{S}^{-}_{3} \cup \mathbb{S}^{-}_{4} \cup \mathbb{S}^{-}_{5}\\ \mathbb{S}^{-}_{i} &\in \mathbb{S}^{\text{U}}_{i} \end{aligned}\end{align*}

where $i \in \{1,2,3,4,5\}$.

The training set and test set can be denoted as follows:


(16) \begin{align*}& \begin{aligned} \mathbb{S}^{\text{train}}_{i} &= \complement_{\mathbb{S}^{+}}{\mathbb{S}^{+}_{i}} \cup \complement_{\mathbb{S}^{-}}{\mathbb{S}^{-}_{i}}\\ \mathbb{S}^{\text{test}}_{i} &= \mathbb{S}^{+}_{i} \cup \mathbb{S}^{\text{U}}_{i} \end{aligned}\end{align*}

where $i \in \{1,2,3,4,5\}$, $\complement $ is the notion of complement operation. It should be noted that the corresponding set for each fold is different, the GIP kernel similarity matrix $\boldsymbol{S}_{p}^{\text{GIP}}$ and $\boldsymbol{S}_{d}^{\text{GIP}}$ needs to be recalculated based on the current training set.

The area under the receiver operating characteristic curve (AUC), the area under the precision-recall curve (AUPR), accuracy, precision, recall, and the F1-score are commonly employed to assess the performance of prediction models when addressing class imbalance problems.

### Comparison with state-of-the-art methods

We compared the performance of DMGAT with seven state-of-the-art models on the same dataset, including NTSHMDA [24], KATZMDA [25], SDLDA [26], AE-RF [27], ABHMDA [28], DMFCDA [29], and DMFMDA [30].

The performance of these models is shown in Table 1. The DMGAT model demonstrates significant advantages in ncRNA-drug association prediction compared to other methods. It achieves the highest AUC (0.8964), AUPR (0.8984), recall (0.9576), and F1-score (0.8285), indicating superior predictive performance. These results suggest that DMGAT excels in overall prediction accuracy and its capacity to handle imbalanced data.

**Table 1:** Performance comparison among different methods

Methods	AUC	AUPR	Recall	F1-score
NTSHMDA	0.7142	0.6391	0.4470	0.5289
KATZMDA	0.7544	0.8048	0.6223	0.6964
SDLDA	0.8258	0.8663	0.6978	0.7588
AE-RF	0.8390	0.8535	0.6127	0.6881
ABHMDA	0.8428	0.8516	0.8413	0.7756
DMFCDA	0.8449	0.8649	0.7463	0.7499
DMFMDA	0.8491	0.8546	0.8288	0.8269
DMGAT	**0.8964**	**0.8984**	**0.9576**	**0.8285**

Numbers in bold indicate the best performance.

Compared to existing models, our model uses sequence information from ncRNA and drugs to extract features, while most other models, such as NTSHMDA, DMFMDA, and SDLDA, use one-hot encoding, ignoring important sequence information. Additionally, sensitivity association helps select more reliable negative samples, effectively addressing the sample imbalance problem. Moreover, the diffusion map dimensionality reduction method enhances feature continuity in the manifold space, which benefits downstream learning. By leveraging these approaches, the GAT graph neural network can better utilize attention mechanisms to fully capture the association features between RNA and drugs, ultimately improving performance.

We also compared the prediction ability of lncRNA and miRNA associated with drug resistance separately with other models. Table 2 shows the prediction ability of DMGAT compared to several other state-of-the-art models for lncRNA and drug association. The data of the performance of other lncRNA-drug prediction models is from [50]. Table 3 shows the prediction ability for miRNA and drug association. The data of the performance of other miRNA-drug prediction models are from [51].

**Table 2:** Performance of lncRNA-drug prediction comparison among different methods

Methods	AUC	AUPR
NetLapRLS	0.811	0.547
BLM-NI	0.784	0.560
KBMF2K	0.841	0.521
CMF	0.834	0.538
DMGAT	**0.8397**	**0.8431**

Numbers in bold indicate the best performance.

**Table 3:** Performance of miRNA-drug prediction comparison among different methods

Methods	AUC	AUPR	Recall	F1-score
CF	0.8618	0.2046	0.3314	0.2873
LP	0.8610	0.2262	0.3176	0.3075
GF	0.8530	0.1619	0.2745	0.2318
SDNE	0.8693	0.1872	0.3012	0.2629
DMGAT	**0.8928**	**0.8961**	**0.9500**	**0.8267**

Numbers in bold indicate the best performance.

### Ablation study

#### Impact of each module

To better evaluate the improvement of each module in our proposed model, we analyze the contributions of various components in the DMGAT model by evaluating versions with specific modules removed. The full DMGAT model achieves the highest performance in terms of AUC and AUPR. When the encoding module was replaced from word2vec to one-hot, TF-IDF (term frequency-inverse document frequency) and BoW (bag-of-words), there is a slight drop in performance. TF-IDF is a numerical statistic that reflects the importance of a word within a document relative to a corpus, by considering its frequency in the document and its rarity across the entire dataset . BoW is a text representation method that models a document as an unordered set of words, disregarding grammar and word order while preserving word frequency information. When the diffusion map feature extraction module is removed, the model’s performance deteriorated. When the GCN module is removed, the similarity matrix between ncRNA and drug is filled into the diagonal position of the adjacency matrix of GAT’s heterogeneous graph, representing the transition of ncRNA and drug with themselves. For the model without a GAT module, directly multiply the features of ncRNA and drug after passing through graph convolution network to obtain the associated prediction score. The GAT module appears to be the most critical component, as its removal causes the largest drop in performance.

While the GCN and diffusion map also contribute to the model’s effectiveness, their impact is less pronounced compared to GAT. The full DMGAT model benefits from the interplay of all three components, yielding the highest scores across all evaluation metrics. As shown in Table 4, the best performance is achieved by combining the three modules.

**Table 4:** Performance comparison among removing different modules of DMGAT

Methods	AUC	AUPR	Recall	F1-score
DMGAT (one-hot)	0.8741	0.8778	0.9577	0.8031
DMGAT (TF-IDF)	0.8629	0.8668	0.9557	0.7952
DMGAT (BoW)	0.8534	0.8605	0.9587	0.7855
DMGAT (no diffusion map)	0.8378	0.8495	0.9633	0.7706
DMGAT (no GCN)	0.8311	0.8416	**0.9655**	0.7641
DMGAT (no GAT)	0.8211	0.8442	0.9533	0.7776
DMGAT	**0.8964**	**0.8984**	0.9576	**0.8285**

Numbers in bold indicate the best performance.

#### Impact of the number of GCN and GAT layers

**Figure 2:**
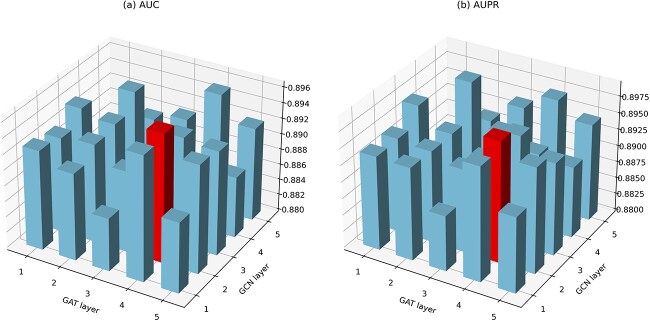
(a) AUC values of DMGAT with different number of GCN and GAT layers, (b) AUPR values of DMGAT with different number of GCN and GAT layers.

To obtain the optimal number of GCN and GAT layers, we iterate the number of GCN and GAT layers from 1 to 5. Fig. 2 shows the AUC and AUPR values of different combination of the number of GCN and GAT layers. The AUC value reaches its peak at 0.8964 when the numbers of GCN and GAT are 2 and 4, respectively, while the AUPR value remains relatively high at 0.8984. So, we choose the model with two layers GCN and four GAT layers.

### Case study

In order to verify if the model can accurately predict new ncRNA-drug associations in the dataset, we used all data to train the parameter and made prediction score for all ncRNA-drug pairs. We selected the top 20 associations with the highest predicted scores of all unlabeled associations. As shown in Table 5, it can be seen that 17 out of the 20 top associations have already been experimentally verified. For instance, miR-26b enhances gemcitabine resistance in the pancreatic cancer cell line PANC-1 by inhibiting p53 expression through targeting the 3UTR of the p53 gene [52]. Similarly, MiR-195 down-regulation promotes 5-fluorouracil (5-FU) resistance in gastric cancer by upregulating HMGA1 expression, thereby contributing to acquired drug resistance [53]. Moreover, Let-7c is down-regulated in gemcitabine-resistant pancreatic cancer cells, contributing to the acquisition of epithelial-to-mesenchymal transition (EMT) characteristics. Restoring let-7c expression can reverse EMT and improve gemcitabine sensitivity, highlighting its potential as a therapeutic target for overcoming drug resistance [54]. Furthermore, MiR-134 is downregulated in cisplatin-resistant lung adenocarcinoma cells, contributing to multidrug resistance (MDR) by targeting forkhead box M1 and multidrug resistance-associated protein 1. Restoring miR-134 expression may improve cisplatin sensitivity and help to overcome MDR in lung adenocarcinoma [55].

**Table 5:** The top 20 predicted scores of ncRNA-drug resistance associations

ncRNA	Drug	Evidence
miR-26b	Gemcitabine	23799850
miR-195	5-Fluorouracil	31115003
miR-26a	Gemcitabine	39288140
miR-200a	Gemcitabine	19654291
miR-365	Doxorubicin	Unconfirmed
miR-30a	Cisplatin	27212164
miR-122	Gemcitabine	31733293
miR-654-5p	Doxorubicin	32329825
miR-423	Doxorubicin	30344696
let-7c	Gemcitabine	19654291
miR-768	Doxorubicin	32714393
miR-4454	Doxorubicin	34777698
miR-23b	Doxorubicin	34216852
miR-133b	Gemcitabine	Unconfirmed
miR-519d	5-Fluorouracil	29771440
miR-216	Gemcitabine	Unconfirmed
miR-196a-5p	5-Fluorouracil	33130965
miR-146b	5-Fluorouracil	29048680
miR-134	Cisplatin	28454276
miR-449a	Cisplatin	24248414

## Discussion

To validate the biological reasonableness of feature extraction in our model, we calculated the cosine similarity of features among ncRNAs associated with the same drug. Each drug is associated with several ncRNAs, and we computed the average cosine similarity between these ncRNAs. A higher value indicates that ncRNAs associated with this drug have more similar features.

**Figure 3:**
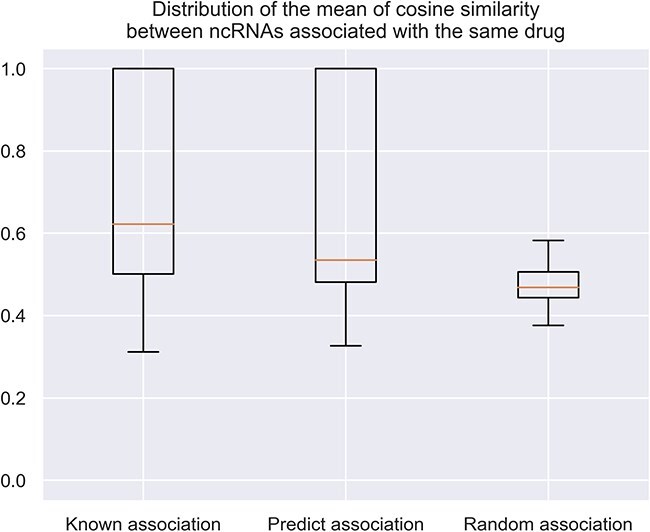
Distribution of the mean of cosine similarity between ncRNAs associated with the same drug.

The distribution of these values for all drugs is shown in Fig. 3. The first column represents the similarity distribution of only known associated ncRNAs, the second column shows the similarity distribution of only predicted associated ncRNAs, and the third column displays the similarity distribution of randomly assigned ncRNAs to drugs, these associations may contain some false associations.

From Fig. 3, the first column has the highest average value, indicating that ncRNAs associated with a drug have very similar features. The second column’s average value is greater than the third column, showing that the features of predicted associated ncRNAs are closer to each other than randomly selected ncRNAs. This demonstrates that the model can effectively distinguish ncRNAs associated with different drugs in the feature space.

The current large-scale validation of case studies is a challenging issue. We have made efforts to validate our method and prediction results from three perspectives.

- Computational evaluation: We constructed a reliable negative sample set and applied computational metrics such as AUC and AUPR to demonstrate the superiority of our method over existing approaches (as shown in Tables 1, 2 and 3).
- Feature similarity analysis: We introduced a new metric, where we assessed that ncRNAs with similar embedding features should be associated with similar drugs (illustrated in Fig. 3).
- Experimental verification: We selected 20 predicted associations not present in the database, 17 of which were subsequently confirmed by other researchers through experimental validation. The number of confirmed predicted associations is higher than that of other methods (as shown in Table 5).

While we have made significant efforts to validate our approach computationally, biological experimental validation remains essential. Most studies analyze the relationship between ncRNA and drug resistance through specific biological models in vivo and in vitro. We look forward to further biological validation of the 3 associations from the top 20 predictions that have not yet been experimentally verified.

Regarding the concern about the difficulty of processing and validating large-scale data, it is indeed not a linear process. As the number of ncRNAs and drugs increases, the computational power and time required for processing and verification also grow significantly. Fortunately, research on ncRNA-drug associations is not yet as mature as that on drug–drug or drug–protein interactions, and the current data scale is still relatively small. In the future, as the data grow, we may consider using techniques such as knowledge distillation to reduce computational overhead.

Our work focuses specifically on predicting ncRNA–drug associations, we did not include ncRNA–disease or drug–disease interaction data in this study. However, there remains uncertainty about the therapeutic relevance of the predictions without the disease context, especially in distinguishing true targeted treatment effects from secondary or off-target associations. For example, BC200 lncRNA is overexpressed in colorectal cancer cells and is located adjacent to the oncogene EpCAM. BC200 RNA and EpCAM are involved in cell migration and invasion. A drug targeting BC200 might influence EpCAM activity, leading to effects that could be either therapeutic or off-target, depending on the context. [56] Integrating disease-level associations is crucial for understanding how ncRNA-drug interactions can translate into clinical outcomes.

However, bridging this gap requires establishing causal links between ncRNAs, drugs, and the biological processes of diseases, and confirming these relationships through large-scale validation. Incorporating additional information under conditions of insufficient disease-related data can increase the complexity of a multimodal model, potentially leading to adverse or counterproductive effects. And also, drug sensitivity and drug resistance prediction tasks also differ in how useful disease context is [57]. An ncRNA’s association with a disease may reflect many disease-related processes or biomarkers unrelated to drug action, so including that data can mislead the model away from true resistance pathways.

Based on the above reasons, we chose not to incorporate disease-level information into our current prediction model. However, if these issues can be addressed in the future, disease information could play a significant supporting role in drug screening and medical diagnosis.

## Conclusion

In this study, a deep learning model called DMGAT using diffusion map was proposed to predict the possibility of ncRNA-drug associations. This model integrated heterogeneous convolution network with heterogeneous graph attention neural network to extract potential features of ncRNA and drug. Additionally, to tackle the imbalance and sparsity problem of dataset, the sensitivity between ncRNAs and drugs are introduced to select reliable negatives.

The performance of DMGAT was evaluated through 5-fold cross-validation on the dataset organized from NoncoRNA and ncDR. DMGAT performs better than the other seven state-of-the-art methods on metrics in terms of AUC, AUPR, recall, and F1-score. Ablation experiments show that all module is essential for achieving that good performance. Then, we conducted a case study on the top 20 associations predicted, which successfully identified differentially expressed ncRNAs from the literature, demonstrating the model’s capability to predict potential correlations and offering valuable insights for future experimental validation.

However, there are still some limitations in our approach. First, the use of word2vec and diffusion map for sequence embedding, while effective, can be computationally intensive for large datasets. As the amount of ncRNA and drug data continues to grow rapidly, this technique may face challenges when dealing with future massive datasets. Second, the attention mechanism in our graph attention network requires a large number of parameters. When dealing with extensive data, this could lead to significant memory usage and increased computational demands, potentially resulting in longer training and inference times. Additionally, our current model only considers the associations between ncRNAs and drugs. In the future, we aim to expand this model by incorporating other relevant information, such as ncRNA-disease associations and drug-disease interactions. This expansion could lead to the development of a more comprehensive model capable of simultaneously predicting the relationships among ncRNAs, drugs, and diseases, thereby providing a more holistic view of these complex biological interactions.

**Key Points**

- We use word2vec and diffusion map to embed the non-coding RNA (ncRNA) sequence and drug simplified molecular input line entry system, which not only includes information of the entire sequence but also information of subsequences.
- Sensitivity associations is applied to select reliable negative associations using a random forest classifier for addressing the problem of imbalanced dataset.
- Attention mechanism of graph network is applied to integrate the feature of ncRNAs and drugs, which can integrate their information throughly.
- The experimental results on benchmark datasets show that diffusion map and heterogeneous graph attention network (DMGAT) outperforms other seven state-of-the art approaches. Case study shows DMGAT has the potential to identify new ncRNA-drug resistance pairs.

## Data Availability

The raw data are released to Zenodo with identifier 13929676. The source code of DMGAT is available at https://github.com/liutingyu0616/DMGAT/tree/main.
